# Exosomes as a Novel Approach to Reverse Osteoporosis: A Review of the Literature

**DOI:** 10.3389/fbioe.2020.594247

**Published:** 2020-10-23

**Authors:** Xudong Xie, Yuan Xiong, Adriana C. Panayi, Liangcong Hu, Wu Zhou, Hang Xue, Ze Lin, Lang Chen, Chenchen Yan, Bobin Mi, Guohui Liu

**Affiliations:** ^1^Department of Orthopedics, Union Hospital, Tongji Medical College, Huazhong University of Science and Technology, Wuhan, China; ^2^Division of Plastic Surgery, Brigham and Women’s Hospital, Harvard Medical School, Boston, MA, United States

**Keywords:** osteoporosis, exosomes, mesenchymal stem cells (MSCs), osteoblasts, osteoclasts

## Abstract

Osteoporosis is a chronic disease requiring long-term, sometimes lifelong, management. With the aging population, the prevalence of osteoporosis is increasing, and with it so is the risk of hip fracture and subsequent poor quality of life and higher mortality. Current therapies for osteoporosis have various significant side effects limiting patient compliance and use. Recent evidence has demonstrated the significant role of exosomes in osteoporosis both *in vivo* and *in vitro*. In this review, we summarize the pathogenesis of senile osteoporosis, highlight the properties and advantages of exosomes, and explore the recent literature on the use of exosomes in osteogenesis regulation. This is a very helpful review as several exosomes-based therapeutics have recently entered clinical trials for non-skeletal applications, such as pancreatic cancer, renal transplantation, and therefore it is urgent for bone researchers to explore whether exosomes can become the next class of orthobiologics for the treatment of osteoporosis.

## Introduction

Osteoporosis is a systemic skeletal disease characterized by low bone mass and decreased bone quality, both of which contribute to an increase in bone fragility (van den Bergh et al., 2012). Fractures are more likely to occur when traumatic forces are exerted on osteoporotic bone, and, hence, osteoporosis is a significant risk factor for fracture. Furthermore, fractures due to osteoporosis are becoming increasingly more common, especially in women over the age of 55 and men over the age of 65, resulting in adverse physical and psychosocial consequences and increased mortality and health-care costs.

Currently available drugs for osteoporosis can be divided into antiresorptive modulators, which include estrogen, estrogen receptor modulators, calcitonin and bisphosphonates, and anabolic therapies, which includes teriparatide. These drugs usually cause adverse effects, and consequently result in decreased adherence. In the case of teriparatide, research has shown that 77% of participants taking 20 μg and 78% of participants taking 40 μg experienced an adverse side effect, including joint pain, muscle cramp, and fatigue (Tsai et al., 2019). Estrogen therapy has known side effects on the breasts and uterus particularly with long-term use. Long-term calcitonin therapy can result in tolerance due to production of circulating antibodies to calcitonin (Wada et al., 1996). Bisphosphonates, the most common current therapy for osteoporosis, result in general side effects including gastrointestinal irritation, bone and joint pain and jaw osteonecrosis (Marx, 2003; Ruggiero et al., 2004). Thus, development of therapies with high bone targeting ability and low toxicity is imperative. Exosomes may be a promising approach to reverse osteoporosis due to their fewer safety considerations as well as powerful pro-osteogenesis abilities.

This review aims to cover the knowledge advances that have been made on the pathogenesis of osteoporosis, the properties of exosomes, and is primarily focused on postmenopausal or senile osteoporosis. We review target cells for the action of exosomes on reversing osteoporosis, and the future for new treatment paradigms for osteoporosis.

## The Pathogenesis of Osteoporosis

### Normal Bone Homeostasis

**FIGURE 1:**
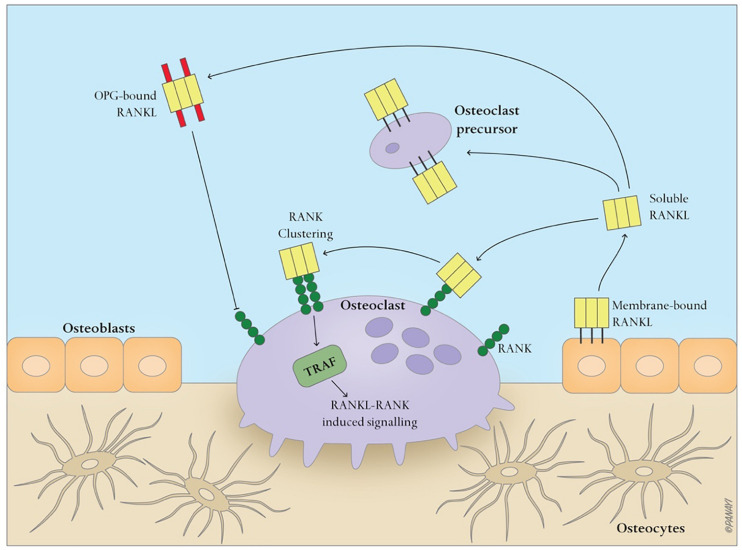
Simplified OPG-RANK-RANKL pathway in bone. RANKL, a homotrimer, is mainly expressed either on the cell membrane of osteoblasts or as a soluble ligand. After RANK binds to RANKL, RANK cluster, recruiting TNFR-associated factors (TRAFs), especially TRAF6, which activate RANK within the cells, and regulate osteoclasts-related gene expression. OPG, a secreted decoy receptor of RANKL, serves as a physiological inhibitor of RANKL-driven osteoclast activities.

**FIGURE 2:**
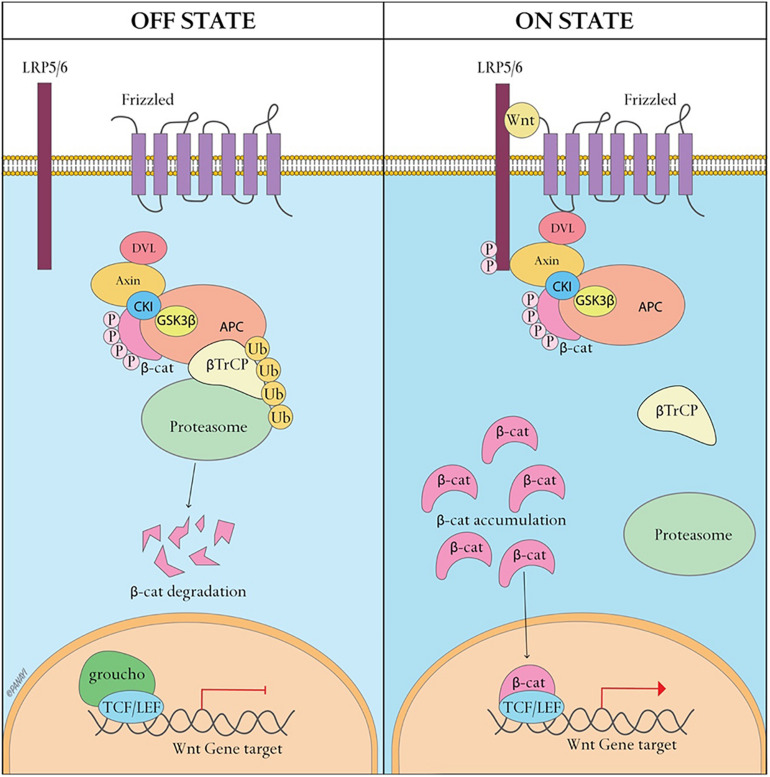
Canonical WNT signaling. A WNT ligand can activate the canonical signaling pathway through binding to a receptor complex formed by low-density lipoprotein (LDL)-related receptor (LRP)5/6 and Frizzled. This results in the translocation of hypophosphorylated β-catenin to the nucleus. In the inactive state, β-catenin is degraded by the proteasome after it becomes phosphorylated by the glycogen synthase kinase 3β(GSK3β)-Axin-casein kinase 1 (CK1)–adenomatous polyposis coli (APC) complex and subsequently ubiquitinated.

Bone is a dynamic tissue that maintains homeostasis through a balance of bone resorption by osteoclasts and bone formation by osteoblasts, through which old bone is replaced with new bone. During this process, osteoclasts are recruited to initiate the resorption of mineralized bone, followed by a reversal phase during which osteoclasts undergo apoptosis. Recruitment of osteoblasts is followed by the formation and mineralization of new bone within the resorption cavity. The sequence of the events is always bone resorption followed by bone formation, and the two processes are tightly coupled, both spatially and temporally. The OPG/RANK/RANKL pathway (Figure 1) has been shown to be associated with osteoclast formation whereas the Wnt/β-catenin (Figure 2) has been shown to play a significant role in osteoblast formation (Lacey et al., 1998). Osteoclasts derived from cells of the myeloid lineage differentiate into pre-osteoclasts which express RANK in the presence of M-CSF and RANKL on the cell membrane of stromal and osteoblast lineage cells. They proliferate and fuse to form bone-resorbing osteoclasts. However, OPG, is a naturally circulating inhibitor/decoy receptor for RANKL, and can bind to RANKL to inhibit osteoclastogenesis and prevent bone resorption (Figure 1) (Simonet et al., 1997; Khosla, 2001). Wnt is the key initiating factor of the Wnt/β-catenin signal pathway. WNT ligands bind to a receptor formed by low-density lipoprotein (LDL)-related receptor (LRP)5/6 and Frizzled (FZD) to activate the signal pathway, and β-catenin is translocated to the nucleus to promote osteoblast formation.

### Pathophysiology of Age-Related Osteoporosis

Briefly, osteoporosis is due to an imbalance between the activities of osteoblasts and osteoclasts. Aging in women is associated with a decline in serum estrogen that has the potential to promote bone formation through inhibition of receptor activator of nuclear factor-κB ligand (RANKL) (Eghbali-Fatourechi et al., 2003), resulting in bone loss and disruption of bone microarchitecture. Trabecular thinning and loss of trabeculae in cancellous bone, cortical thickness and increased cortical porosity in cortical bone have been noted in older women (Parfitt et al., 1983; Compston et al., 1987; Zebaze et al., 2010). In men, aging plays a predominant role in osteoporosis, resulting in decreased bone formation through reduced proliferation and differentiation of multipotent stem cells (MSCs) and decreased bone matrix production depended on decreased osteoblast function and increased apoptosis of mature osteoblasts (Manolagas and Parfitt, 2010). Cell-to-cell communication is becoming increasingly accepted as a mechanism impacting the development of osteoporosis. A therapy that affects the activities of MSCs, osteoblasts and osteoclasts by influencing intercellular communication would, therefore, be a potential future treatment. Given that exosomes, which are extracellular vesicles, have this ability, we set out to investigate these vesicles in depth.

## The Properties of Exosomes

**FIGURE 3:**
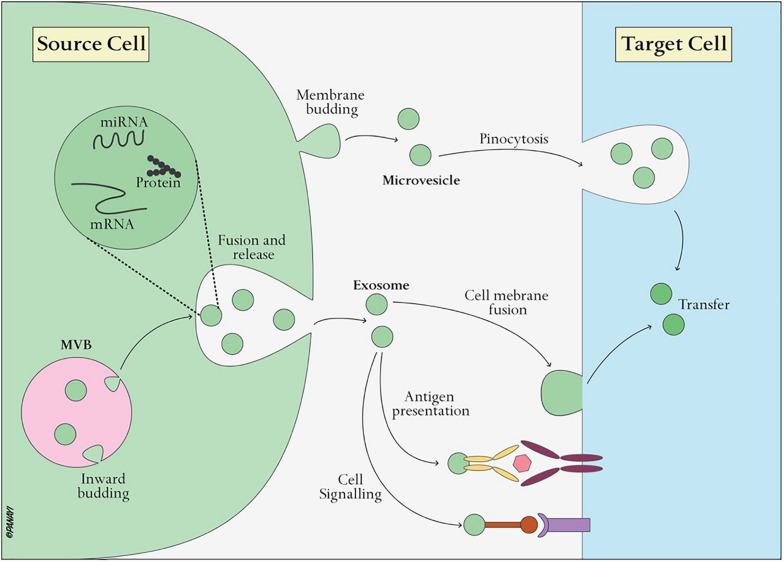
Exosome biogenesis and interaction with target cells. Exosomes are vesicles of endocytic origin formed by the inward budding of the multivesicular body (MVB) membrane. Exosomes can then directly activate cell surface receptors via protein and bioactive lipid ligands, transfer cell surface receptors or deliver effectors including transcription factors, oncogenes and infectious particles into target cells. In addition, various RNA species including mRNAs and small regulatory RNAs such as microRNAs (miRNAs) and non-coding RNAs are contained in extracellular vesicles and delivered to target cells.

Exosomes, nanoscale vesicles ranging from 30–120 nm in diameter (Table 1) (EL Andaloussi et al., 2013b), are extracellular vesicles widely distributed in almost all biological fluids, including plasma, urine, saliva, ascites, amniotic fluid, lactic acid and cerebrospinal fluid (Simons and Raposo, 2009; Gross et al., 2012). Almost all cells, including B cells (Raposo et al., 1996), T cells (Blanchard et al., 2002), dendritic cells (Thery et al., 1999), mast cells (Raposo et al., 1997), and endothelial cells (Song et al., 2019), can secrete exosomes. Exosomes, as intraluminal endosomal vesicles, are released by exocytosis when multivesicular bodies (MVBs) fuse with the plasma membrane (Figure 3) (Heijnen et al., 1999). The exosome membrane is mainly composed of lipids and proteins, and the exosomal lumen is enriched with bioactive molecules, including proteins, lipids, metabolites, mRNA, microRNA (miRNA), and other non-coding RNA (ncRNA) (Gross et al., 2012; Sato-Kuwabara et al., 2015). Exosomes directly activate cell surface receptors through protein and bioactive lipid ligands, and initiate fusion of their membrane contents with the recipient cell plasma membrane, playing a significant role in regulating the bioactivity of recipient cells by conveying lipids, proteins and nucleic acids. In this way, exosomes are not only involved in the maintenance of normal physiology, such as stem cell maintenance (Mehta et al., 2015), tissue repair (Li et al., 2013), organ development, hematopoietic function, but also participate in diseases processes, such as cancer metastasis and angiogenesis (Al-Nedawi et al., 2008).

**TABLE 1:** Exosome, microvesicle, apoptotic body: major differences.

	Vesicle types		
Characteristics	Exosomes	Microvesicles	Apoptotic bodies
Size	30–120 nm	50–1,000 nm	500–2,000 nm
Morphology	Cup-shaped	Heterogeneous	Heterogeneous
Origin	Endolysosomal pathway; multivesicular bodies	Plasma membrane	Apoptotic cell membrane
Marker	Tetraspanins, ESCRT Components, TSG101, ALIX	Integrins, selectins, CD40	Phosphatidylserine
Mechanism of discharge	Exocytosis of multivesicular bodies	Outward budding of plasma membrane	Outward blebbing of apoptotic cell membrane
Contents	Protein, miRNA, mRNA, non-coding RNAs	Protein, miRNA, mRNA, non-coding RNAs	Nuclear fractions, cell organelles

**ESCRT, endosomal sorting complex required for transport; TSG101, tumor susceptibility gene 101 protein; ALIX, programmed cell death 6 interacting protein (also known as PDCD6IP)*.*

Among their cargoes, miRNAs in the exosomal lumen have garnered increasing attention. MiRNAs, a class of 17–24 nt non-coding RNAs, play a regulatory role in recipient cells through targeting mRNAs for cleavage or translational repression (Bartel, 2004). In addition, some miRNAs, such as tumor-secreted miRNA-21 and miRNA-29a, have the capacity to act as ligands that bind to toll-like receptors (TLRs) and then activate immune cells (Fabbri et al., 2012). Additionally, lncRNAs, non-coding RNAs with a length of more than 200 nucleotides (Ulitsky and Bartel, 2013), are known to be involved in various biological processes, and have important roles in bone formation and regeneration (Wang et al., 2015). Currently, it is recognized that lncRNAs act mainly through regulation of RNA and protein through interactions between RNA and RNA or RNA and DNA (Li Y. et al., 2016).

In addition to naturally secreted exosomes, however, genetically engineered or chemically modified exosomes which enable cell-specific delivery of cargoes have been studied in recent years (Armstrong et al., 2017). For example, chondrocyte-specific dendritic cell-derived exosomes modified through genetic engineering successfully achieved targeted delivery of miRNA-140 to chondrocytes in deep regions of the cartilage. This was shown to improve the osteoarthritic cartilage (Liang et al., 2020). Treatment with iExosomes (engineered exosomes) in mice with pancreatic cancer significantly increased overall survival through targeting oncogenic KRAS (Kamerkar et al., 2017). Hence, conventional drug delivery vehicles have disadvantages that are largely absent when exosomes are used as drugs carriers, including potential toxicity, immunogenicity and inability to penetrate target specific organs.

## Exosomes Regulate Mesenchymal Stem Cell Osteogenic Differentiation

Mesenchymal stem cells (MSCs), non-hematopoietic multipotent stem cells, have multilineage differentiation potential including differentiation into osteoblasts, chondrocytes and adipocytes (Caplan, 1991). MSCs play a significant role in maintaining the homeostasis between bone resorption and bone formation.

### Exosome-Mediated Differentiation of MSCs

Exosomes are important vesicles in intercellular communication for modulating or mediating cellular processes. Therefore, exosomes can direct MSCs differentiation toward osteoblastic lineage. For instance, exosomes secreted by MSCs derived from human induced pluripotent stem cells (hiPSCs, hiPSC-MSC-Exos) effectively promote the proliferation and osteogenic differentiation of BMSC *in vitro*. There was a dose-response relationship between the effect and exosomes concentration (Qi et al., 2016). Similarly, one study suggested that exosomes derived from osteogenic MSCs can direct undifferentiated MSCs toward osteogenic differentiation both *in vivo* and *in vitro* (Narayanan et al., 2016). Furthermore, osteoblast-derived exosomes can increase osteogenic differentiation by enhancing osteogenic gene expression in MSCs (Narayanan et al., 2018), establishing a positive feedback of bone formation or growth. In addition, exosomes derived from monocytes have been proven to be stimulatory on MSCs osteogenic differentiation *in vitro* (Gebraad et al., 2018). Among them, miRNA in exosomes has been shown to be a critical component in stem cell differentiation (Shim and Nam, 2016). Nine exosome-derived miRNAs (including let-7a, miRNA-199b, miRNA-218, miRNA-148a, miRNA-135b, miRNA-203, miRNA-219, miRNA-299-5p, and miRNA-302b) have been shown to be significantly up-regulated and three exosome-derived miRNAs (miRNA-885-5p, miRNA-181a, and miRNA-320c) showed significant down-regulation (Xu et al., 2014). Additionally, some studies have shown that miRNA-138a, miRNA-141 and miRNA-200a can inhibit MSCs differentiation into osteoblasts (Itoh et al., 2009; Eskildsen et al., 2011). In addition to miRNA, lncRNA might be involved in the process of osteogenic differentiation of MSCs, such as lncRNA H19 (Wang et al., 2017). Although MSCs osteogenic differentiation is regulated by exosomes, it cannot be concluded which cell type-derived exosome was dominant.

### Possible Regulatory Mechanism

Exosomes are more likely to play an important role in regulating MSCs differentiation through their non-coding RNA (miRNA and lncRNA), and thus affect expression of some transcription factors. Differentiation of MSCs toward osteocytes is regulated by various transcription factors, such as Runt-related transcription factor 2 (Runx2), osterix, β-catenin, and bone morphogenetic proteins (BMPs), whose expression is regulated by many signaling pathways, such as essential Wnt, BMP. The Runx2 transcription factor is an essential regulator for the osteogenic differentiation of MSCs, which can induce differentiation of MSCs into pre-osteoblasts and inhibits adipogenic and chondrogenic differentiation (Komori, 2006). For example, miRNA-133 can inhibit differentiation of MSCs into osteoblasts by down-regulation of the expression of Runx2 (Li et al., 2008). Osterix, a specificity protein 1 family (Sp1) of transcription factors, is downstream of Runx2, and is essential for bone formation (Nakashima et al., 2002). For example, lncRNA MALAT1 stimulates osterix expression and osteogenesis of MSCs by sponging miRNA-143 (Gao et al., 2018), and the lncRNA-miRNA pathway is the most classic mechanism of action. In addition, some miRNAs regulate MSCs differentiation through indirect interaction with transcription factors, and lncRNA regulates differentiation of MSCs through others pathways, including Wnt/β-catenin pathway, p38 MAPK pathway, and BMP4 signaling (Ju et al., 2019).

### Exosomes Acting on MSCs Reverse Osteoporosis

Stem cell therapies have shown great potential in the treatment of various diseases, and as exosomes carry a cargo of functional signals in the form of RNA, miRNA, or protein, they can act as pivotal messengers of intercellular communication. Therefore, exosomes treatment may offer similar potential as stem cell transplantation. For example, exosomes originating from exogenous MSCs can promote osteogenic differentiation of endogenous MSCs and improve osteoporosis (Liu et al., 2015). Exosomes from endogenous BMSCs can target endogenous MSCs to enhance osteogenic differentiation and reduce adipogenic differentiation, while miRNA-151-5p administered alone also achieves the same effect (Chen et al., 2017). Additionally, a study demonstrated that normal MSCs-derived extracellular vesicles (containing rich exosomes) can promote proliferation and reduce DNA damages of MSCs after radiation exposure (Wen et al., 2016). Thus, intravenous injection of MSCs-derived exosomes to patients with osteoporosis is a promising therapy.

## Exosomes Regulate Osteoblast Proliferation and Activity

Approximately 4–6% of the total resident cells in bone are osteoblasts, which are essential for the growth and maintenance of the skeleton. Additionally, osteoblasts are responsible for synthesis and mineralization of bone matrix, which increase the bone mineral density and prevent fragility fractures.

### Exosomes Participate in Osteoblast Proliferation and Activity

Exosomes can affect osteoporosis by directly regulating osteoblast proliferation and activity. Exosomes derived from different cells or cells with different functional status might have significantly different effects on osteoblasts. MSCs-derived exosomes can promote bone formation *in vivo* through stimulation of osteoblast differentiation, activity and proliferation mainly through miRNA-196a (Qin et al., 2016). Osteoclast-derived exosomes were shown to decrease bone formation through inhibition of osteoblast activity through miRNA-214-3p (Li D. et al., 2016). One study, which found elevated miRNA-214 levels in aged postmenopausal women and aged men, showed that miRNA-214 directly targets ATF4 to inhibit osteoblast activity and antagomirna-214 can promote osteoblast activity and matrix mineralization *in vitro* (Wang et al., 2013). Interestingly, although patients with tumors usually exhibit osteoporosis, exosomes originating from tumors can stimulate osteoblast proliferation and activity. Osteoblasts treated with exosomes derived from breast cancer cells, decreased in number, metabolic activity, and alkaline phosphatase (ALP) activity (Loftus et al., 2020). Similarly, prostate cancer cell-derived exosomes increases osteoblast proliferation by 1.5-fold (Inder et al., 2014).

### Possible Regulatory Mechanism

Although the specific mechanism has yet to be defined, some recognized pathways might be involved. Firstly, proteins on the exosomal membranes, such as cell recognition molecules, can facilitate selective targeting and uptake by target cells. Exosomes entering the osteoblasts through endocytosis, transpor luminal components into the cytosol of the target cell. Finally, some RNAs, such as miRNA, lncRNA, translocate into the nucleus and regulate expression of osteoblast-related genes. For example, the miRNA-214 in osteoclast-derived exosomes could be transferred into osteoblasts through EphrinA2/EphA2 recognition (Sun et al., 2016). MiRNA-214 directly targets activating transcription factor 4 (ATF4) by binding to its 3′untranslated region (UTR) (Wang et al., 2013). Thus, the exosomes inhibit osteoblast proliferation and activity by significantly decreasing bone formation marker gene expression, including expression of osteocalcin (BGLAP) and alkaline phosphatase (ALP).

### Exosomes Undergoing Modification or Material Loading Can Reverse Osteoporosis

Exosomes undergoing modification or material loading have shown great promise for the treatment of osteoporosis. Prior research has shown that exosome-integrated titanium oxide nanotubes can stimulate bone regeneration by promoting osteogenic differentiation (Wei et al., 2019). Similarly, self-assembly of biotinylated MSCs-EVs onto the surface of biotin-doped polypyrrole titanium (Bio-Ppy-Ti) exhibits enhanced osteoinductive ability *in vivo* and *in vitro* through stimulation of osteogenic differentiation, proliferation and activity (Chen et al., 2019). Remarkably, some materials *in vivo* might serve as a bioreactor, which can affect exosome or growth factor production. For example, a recent animal study suggested that magnetic hydroxyapatite (MHA) scaffolds are capable of altering osteoclast-derived exosomal cargoes and decreasing the efficiency of exosome uptake by osteoblasts, further promoting osteoblast proliferation and reversing osteoporosis (Zhu et al., 2020). This evidence suggests that osteoporotic patients with fractures may be treated with materials integrated in exosomes which promote bone formation and reverse osteoporosis. However, more evidence is needed given the scarcity of knowledge on this subject.

## Exosomes Regulate Osteoclast Maturation and Activity

Osteoclasts, multinucleated giant cells formed by the fusion of mononuclear progenitors of the monocyte/macrophage family, are the exclusive cells of bone resorption in the human body, originating from haematopoietic progenitors (Xing et al., 2005). In the process of osteoclastogenesis, pre-osteoclasts (preOCs) are formed first.

### Exosomes Affect Osteoclast Differentiation and Activity

Research has shown that osteoclast activity can determine the development of osteoporosis (Teitelbaum, 2000). Exosomes, as an important intercellular messenger, play a significant role in regulating osteoclast maturation and activity. First, the bone system itself is the most significant regulator of osteoclast differentiation. For example, exosomes from pre-osteoclasts can promote osteoclast formation, whereas exosomes from osteoclasts are pro-inhibitory (Huynh et al., 2016). Exosomes from osteoblasts can facilitate the differentiation of pre-osteoclasts to osteoclasts through bioactive molecular content (RANKL) (Deng et al., 2015). However, osteoclast-derived exosomes inhibit osteoclast formation (Xie et al., 2017). Such evidence suggests that exosomes might participate in intercellular communication among the different types of bone cells. In addition, a recent study has demonstrated that compared to exosomes derived from osteoblasts and BMSCs, endothelial cell-derived exosomes show more efficient bone targeting that suppresses osteoclast differentiation and activity through miRNA-155 (Song et al., 2019). In addition, tumor cells have been shown to increase the number and activity of other cells. Breast cancer cell-derived exosomes can promote the proliferation and differentiation of osteoclasts through miRNA-20a-5p (Guo et al., 2019), and prostate cancer cell-derived exosomes exhibit strong promotion of osteoclastogenesis *in vitro* (Inder et al., 2014). This may contribute to the inherent mechanism through which patients with tumors exhibit osteoporosis. Multiple myeloma-derived exosomes can also stimulate osteoclastogenesis via abundant EGFR ligand amphiregulin (AREG) (Raimondo et al., 2019). Additionally, other tumors, such as non-small cell lung cancer (NSCLC), colon cancer, are rich sources of AREG (Taverna et al., 2017).

### Possible Regulatory Mechanism

According to prior research, the most important signaling pathway regulating osteoclast differentiation and maturation is the RANK/RANKL signaling pathway. The mitogen-associated protein kinase (MAPK) signaling pathways, including p38, extracellular signal-regulated kinase (Erk), Jun N-terminal kinase (JNK), c-Fos and AP-1, also participate in osteoclast formation (Matsumoto et al., 2000; Keshet and Seger, 2010). Thus, these may be possible mechanisms through which exosomes regulate osteoclast differentiation and activity. Notably, exosomes cargoes components, including protein, miRNA, lncRNA, may all play a regulatory role in osteoclast differentiation and activity. AREG, a tumor-derived exosome, can activate the epidermal growth factor receptor (EGFR) pathway in pre-osteoclasts to increase RANKL expression. LncRNA-MALAT 1 from endothelial progenitor cells (EPCs) promote osteoclastogenesis through directly binding to miRNA-124 (Cui et al., 2019). Expression of NFATc1, a downstream signal of RANK, is suppressed by miRNA-124 (Lee et al., 2013). MiRNA-155 in endothelial cell-derived exosomes disrupts osteoclast differentiation and activation through targeting several essential transcription factors, including Spi1, microphthalmia-associated transcription factor (Mitf), and suppressor of cytokine signaling 1 (Socs1) (Song et al., 2019).

### Exosomes Inhibiting Osteoclast Differentiation and Activity Are Predicted to Treat Osteoporosis

Clinical treatment of osteoporosis typically focuses on inhibiting the excessive activation of osteoclasts and exosomes might be a promising candidate for this in the future. Accumulating evidence has shown that exosomes strongly inhibit osteoclast differentiation and activity, effectively reversing osteoporosis *in vitro* and in animal studies (Song et al., 2019). In comparison with other exosomes, endothelial cell-derived exosomes show superiority in targeting bone. Remarkably, exosomal miRNA is thought to be an important component in gene expression regulation participating in the differentiation and activity of osteoclasts, such as endothelial cell-derived exosomal miRNA-155 (Song et al., 2019), miRNA-503 (Chen et al., 2014), miRNA-133a (Wang et al., 2012), and miRNA-422a (Cao et al., 2014), which may offer potential for developing future drugs against osteoporosis. Clinic trials are, however, required to assess the effect of these exosomes on the human body.

## Advantages of Exosomal Treatment

Compared to conventional therapy, exosomal therapy has several advantages. First, exosomes have low immunogenicity. Human marrow-derived MSCs express MHC I molecules on their cell surface, resulting in immunological rejection. Animal studies, however, have shown that MSCs-exosomes can promote bone formation without any adverse events (Zhang et al., 2016). Further, exosomes have low toxicity. In recent years, pharmaceutical carriers, especially nanoscale pharmaceutical carriers, have received extensive attention, but their use is limited due to their synthetic lipid membranes resulting in unavoidable toxic reactions. Exosomes, as natural endogenous nano-microvesicles, have not been shown to induce toxicity neither *in vivo* nor *in vitro* trials (Narayanan et al., 2016; Qin et al., 2016). In addition, exosomes offer excellent targeting. For example, osteoclast-exosomes can target osteoblasts through their EphrinA2 (Sun et al., 2016). Exosomes also display great permeability, with studies showing that exosomes can penetrate cytomembranes and biological barriers (El Andaloussi et al., 2013a; Janas et al., 2015). Furthermore, studies have highlighted their excellent stability; PTX (Paclitaxel) incorporated into exosomes by mild sonication can maintain stability at various conditions for over a month (Kim et al., 2016). Finally, their function can be enhanced through modification or material loading. Given the aforementioned advantages, exosomes have strong potential to treat osteoporosis through bone tissue engineering and as targeted delivery vehicles. In bone tissue engineering, exosomes loaded with materials offer unique advantages in patients with osteoporosis following a fracture. Exosomes, as targeted delivery vehicles, can also effectively load compounds such as miRNA, and siRNA.

## Clinical Perspective: Still a Way to Go

Although some clinical trials have been completed, there are currently no clinically approved therapies utilizing exosomes. This is largely due to lack of knowledge on the fundamentals of exosome biology and methodology. It should be noted, for example, that the specimen from which exosomes are to be harvested determines their potential as regulators. A decision must also be made whether to choose autografts or allografts. Another challenge is the limited ability of current isolation methods resulting in only low yield and low-purity exosomes. For example, 5 × 10^6^ myeloma cells provide only 5–6 μg of exosomes (Peinado et al., 2012). In addition, current isolation methods of exosomes include mainly repeated ultracentrifugation and ultrafiltration (Lobb et al., 2015), techniques which are too time-consuming and remain somewhat controversial. Another major challenge is that the exact function of the cargoes in exosomes has yet to be established. For example, cargoes from the same cells derived from different tissues or organs are likely to differ. Research has shown that exosomes derived from bone marrow and adipose tissue differ vastly in tRNAs, especially for Sox2, POU5F1A/B, and Nanog (Baglio et al., 2015). Furthermore, some contents in the exosomes, such as tumor-supportive miRNA found in MSCs-exosomes, might play a role in disease promotion (Vallabhaneni et al., 2015). Therefore, thorough understanding of how to best utilize the function of exosomes in treating osteoporosis still has a way to go.

## Conclusion

Overall, exosomes are natural membrane vesicles involved in intercellular communication by conveying their cargoes, which include proteins, as well as coding and non-coding RNA. Studies have shown that exosomes participate in bone homeostasis through regulation of the differentiation and activity of bone cells including MSCs, osteoblasts and osteoclasts. Thus far their regulatory mechanisms have not been fully elucidated. Given their several intrinsic advantages, such as their low immunogenicity and toxicity, exosomes offer promising therapeutic potential and are expected to become a reliable new approach to treating osteoporosis. Finally, further efforts must be made to improve the isolation and purity methods, as well as gain a better understanding on the roles that exosomes play in the regulation of osteoporosis.

## Author Contributions

XX, YX, and LH proposed the research questions, developed the protocol, and drafted the manuscript. XX guarantor. XX, WZ, and HX refined the search strategy. XX, ZL, LC, and CY searched and collected the studies. AP created the illustrations in Adobe Illustrator 2020. YX, BM, and GL critically reviewed the manuscript for relevant intellectual content. All authors have read and approved the final version of the manuscript.

## Conflict of Interest

The authors declare that the research was conducted in the absence of any commercial or financial relationships that could be construed as a potential conflict of interest.
